# Seminal Plasma Protein N-Glycan Peaks Are Potential Predictors of Semen Pathology and Sperm Chromatin Maturity in Men

**DOI:** 10.3390/life11090989

**Published:** 2021-09-20

**Authors:** Tihana Maric, Ana Katusic Bojanac, Ana Matijevic, Marcello Ceppi, Marco Bruzzone, Evangelini Evgeni, Tea Petrovic, Iwona Wójcik, Irena Trbojevic-Akmacic, Gordan Lauc, Davor Jezek, Aleksandra Fucic

**Affiliations:** 1 Scientific Centre of Excellence for Reproductive and Regenerative Medicine, University of Zagreb School of Medicine, 10000 Zagreb, Croatia; ana.katusic@mef.hr (A.K.B.); davor.jezek@mef.hr (D.J.); afucic@imi.hr (A.F.); 2 Department of Medical Biology, University of Zagreb School of Medicine, 10000 Zagreb, Croatia; 3 Department of Laboratory Diagnostics, University Hospital Zagreb, 10000 Zagreb, Croatia; matijevicana@gmail.com; 4 Clinical Epidemiology Unit, IRCCS Ospedale Policlinico San Martino, 16132 Genova, Italy; marcello.ceppi@hsanmartino.it (M.C.); marco.bruzzone@hsanmartino.it (M.B.); 5 Cryogonia Cryopreservation Bank, 11526 Athens, Greece; info@cryogonia.gr; 6 Genos Glycoscience Research Laboratory, 10000 Zagreb, Croatia; tpetrovic@genos.hr (T.P.); iwojcik@genos.hr (I.W.); iakmacic@genos.hr (I.T.-A.); glauc@genos.hr (G.L.); 7 Department of Biochemistry and Molecular Biology, Faculty of Pharmacy and Biochemistry, University of Zagreb, 10000 Zagreb, Croatia; 8 Department of Histology and Embryology, University of Zagreb School of Medicine, 10000 Zagreb, Croatia; 9 Institute for Medical Research and Occupational Health, 10000 Zagreb, Croatia

**Keywords:** male infertility, sperm, seminal plasma, N-glycosylation, DNA fragmentation, Halosperm, sperm chromatin maturity, aniline blue

## Abstract

Background: Male infertility is increasingly becoming a health and demographic problem. While it may originate from congenital or acquired diseases, it can also result from environmental exposure. Hence, the complexity of involved molecular mechanisms often requires a multiparametric approach. This study aimed to associate semen parameters with sperm DNA fragmentation, chromatin maturity and seminal plasma protein N-glycosylation. Methods: The study was conducted with 166 participants, 20–55 y old, 82 normozoospermic and 84 with pathological diagnosis. Sperm was analyzed by Halosperm assay and aniline blue staining, while seminal plasma total protein N-glycans were analyzed by ultra-high-performance liquid chromatography. Results: Sperm DNA fragmentation was significantly increased in the pathological group and was inversely correlated with sperm motility and viability. Seminal plasma total protein N-glycans were chromatographically separated in 37 individual peaks. The pattern of seminal plasma N-glycan peaks (SPGP) showed that SPGP14 significantly differs between men with normal and pathological semen parameters (*p* < 0.001). The multivariate analysis showed that when sperm chromatin maturity increases by 10%, SPGP17 decreases by 14% while SPGP25 increases by 25%. Conclusion: DNA integrity and seminal plasma N-glycans are associated with pathological sperm parameters. Specific N-glycans are also associated with sperm chromatin maturity and have a potential in future fertility research and clinical diagnostics.

## 1. Introduction

Male infertility, defined as the inability of a fertile female partner to achieve pregnancy, affects approximately 7% of men worldwide and has risen globally in the past few decades [1,2]. In infertile couples, the male factor alone or combined with the female factor contributes to about 40–50% of the cases. Male infertility has a very heterogeneous background and may originate from congenital or acquired conditions, including anomalies of the reproductive system (varicocele, cryptorchidism, hypospadias, etc.), urogenital infections, hormonal disorders, or various genetic aberrations [3]. Environmental stressors can also significantly contribute to the reduction of male fertility through occupational settings such as exposure to hazardous substances, stress, etc., or poor choice of lifestyle habits involving excessive alcohol consumption, smoking and a high-energy diet [4,5].

Infertility is traditionally diagnosed by standard laboratory semen analysis that determines sperm count, motility, viability and morphology [6]. Although semen analysis is considered the cornerstone of the initial evaluation of male fertility parameters, it is frequently not empowered to determine a man’s fertility status. Moreover, its inadequacy in explaining and understanding subtle mechanistic changes in cellular or seminal characteristics that differentiate fertile from infertile subjects requires further development of diagnostic approaches [7,8]. This is especially important for assisted reproductive technology (ART) where prognostic markers of the successful outcome are not always correlated with the ones that define male fertility potential and require additional seminal analysis with more complex prediction models [9].

Seminal plasma is composed of various biomolecules relevant for reproduction, namely fructose as a main dietary source for sperm, extracellular vesicles, proteins, lipids, cell-free DNA, different RNA molecules, and ions. Hence, seminal plasma is a potential source of novel biomarkers related to reproductive disorders [10,11]. N-linked protein glycosylation is a process of attachment of sugar moieties to the amide side chain of asparagine residues (in evolutionary conserved sequence asparagine-any amino acid except for proline-serine/threonine) and is one of the most prevalent post-translational protein modifications with a role in mediation interactions between cells and proteins [12]. Sperm and seminal plasma are rich in glycosylated proteins indispensable for various functions during spermatogenesis, sperm maturation, capacitation and in sperm-egg interaction that finally leads to fertilization [13]. The contribution of seminal plasma glycoproteins to fertilization is mainly to maintain sperm in a decapacitated state until they reach eggs and to assist sperm during transportation in the female reproductive tract [14].

Recently, several studies analyzed the N-glycome of the human spermatozoa and seminal plasma [15,16,17,18]. Pang et al. in 2007 showed that human sperm N-glycome expresses biantennary bisecting type N-glycans [18]. Furthermore, the same researchers detected three major families of N-glycans: high mannose glycans, bi-, tri-, and tetra-antennary core-fucosylated complex type N-glycans, and bi-, tri-, and tetra-antennary core-fucosylated complex type N-glycans with antennae capped with sialic acid. They also confirmed the presence of high mannose and polyfucosylated N-glycans in seminal plasma [15]. The majority of detected N-glycans were complex type, followed by high-mannose and hybrid type, whereas most of the glycans were either sialylated, fucosylated, or both. Importantly, the differences in the glycosylation pattern in men with abnormal semen parameters have been reported in several studies [19,20,21,22,23].

Genome damage and sperm DNA integrity are important factors that influence sperm quality, and the subsequent fertilization and success of IVF procedures [24]. Stressors that induce sperm DNA fragmentation (SDF) are smoking, alcohol, heat exposure, various environmental pollutants, some drugs, and diseases that promote apoptosis and oxidative stress [25]. Assays that measure SDF can further contribute to the evaluation of male infertility [26]. Sperm DNA damage is commonly evaluated with the Halosperm assay that measures SDF by sperm chromatin dispersion [27].

The crucial aspect of healthy spermatozoa development occurs during spermatid elongation where histones wrapped around DNA are sequentially replaced with testis-specific histone variants, transition nuclear proteins, and protamines. This process, also called spermiogenesis, assures proper condensation and termination of transcription of haploid DNA in the spermatid head [28]. Correct DNA packaging and remaining histone levels can have a profound influence on sperm quality and early stages of embryonic development [29]. Thus, several straightforward methods are applied for the evaluation of remaining histones and sperm nuclear condensation during infertility management, including aniline blue (AB) staining, a common assay based on AB affinity for histones. The higher degree of stained sperm cells indirectly demonstrates histone levels and protamination defects critical for DNA condensation and maturation [30]. This biomarker is suggested as an additional parameter in human male fertility assessment [31].

As the role of sperm DNA fragmentation, chromatin maturation and seminal plasma glycosylation is often separately studied in male reproductive health, it is important to profile these parameters simultaneously during infertility estimation in order to get insight into the crossroads of their pathways. Thus, in the present study we aimed to investigate for the first time their association in normozoospermic men and men with pathological semen parameters.

## 2. Results

### 2.1. Descriptive Characteristics of Study Participants

Study participants’ characteristics, including mean values of age, basic sperm parameters, SDF expressed as sperm DNA fragmentation index (DFI), and percentage of sperm chromatin maturity are presented in Table 1 and Table 2. Table 1 shows a statistical comparison between men with normozoospermic (N) and pathological (P) semen parameters. As participants were further divided into pathological sub-groups according to the WHO limits for basic sperm parameters [32], Table 2 presents a comparative analysis of normozoospermic (N) and groups with disrupted semen parameters–asthenozoospermia (A), oligozoospermia (O), and oligoasthenozoospermia (OA). Participants’ age and semen volume did not significantly differ between the N and P nor between the sub-groups.

### 2.2. Association of Sperm DNA Fragmentation and Semen Parameters

**Figure 1:**
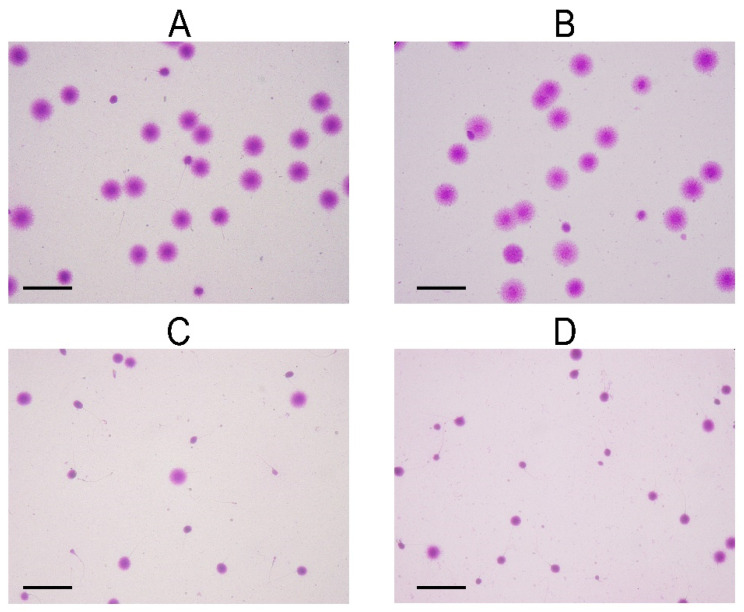
Representative microscopic image of sperm after Halosperm assay for evaluation of DNA fragmentation. (**A**,**B**) Non-fragmented sperm and (**C**,**D**) Severely fragmented sperm. Magnification 400×, scale bar shows 20 µm.

Sperm genome damage, determined by Halosperm assay, is shown as DFI assessed by CASA. The DNA fragmentation index was significantly increased in all groups with pathological semen parameters (30.7 ± 17.5%) (Table 1 and Table 2, Figure 1), compared to the DFI of normozoospermic men (17.8 ± 9.2%). The most significant deviation from the control value of DFI was detected in OA samples (38.9 ± 20.3%), followed by the A (27.6 ± 15.3%) and O (21.6 ± 7.2%) groups (Table 2). Furthermore, sperm halos’ diameters were in concordance with the values of DNA fragmentation (Table 2).

An association was determined between DFI and pathological semen parameters. An inverse correlation was found between DFI and sperm motility and the percentage of viable sperm cells (−0.509). Moreover, the log-normal regression model, adjusted by age, diagnosis, and sperm chromatin maturity, confirmed the correlation between DFI and sperm motility and viability, by showing that sperm motility and viability increase by 10% and lead to sperm DFI decrease by 12.4% (*p* < 0.001) and to sperm DFI reduction by 16.8% (*p* < 0.001), respectively. The results remained significant after Bonferroni’s correction.

### 2.3. Association of Sperm Chromatin Maturity and Semen Parameters

**Figure 2:**
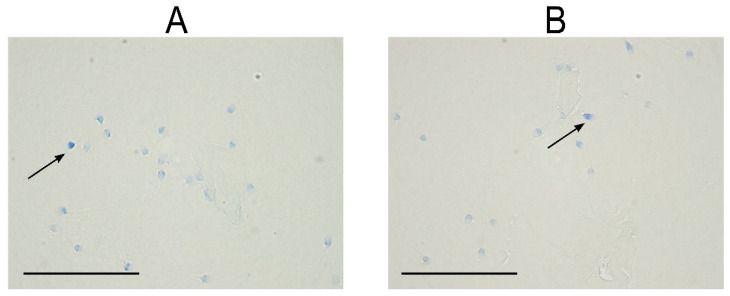
Representative microscopic image of sperm after aniline blue staining for assessment of sperm chromatin maturity of (**A**) normozoospermic men and (**B**) men with pathological semen parameters. Arrows indicate immature dark-blue stained sperm. Magnification 1000×, scale bar shows 50 µm.

Statistical analysis did not show any significant differences in sperm chromatin maturity between N and P or between the other pathological sub-groups (Table 1 and Table 2, Figure 2). Likewise, correlation analysis of sperm parameters and sperm chromatin maturity did not show any significant results.

### 2.4. N-Glycan Composition in Normozoospermic and Pathological Diagnosis

**Figure 3:**
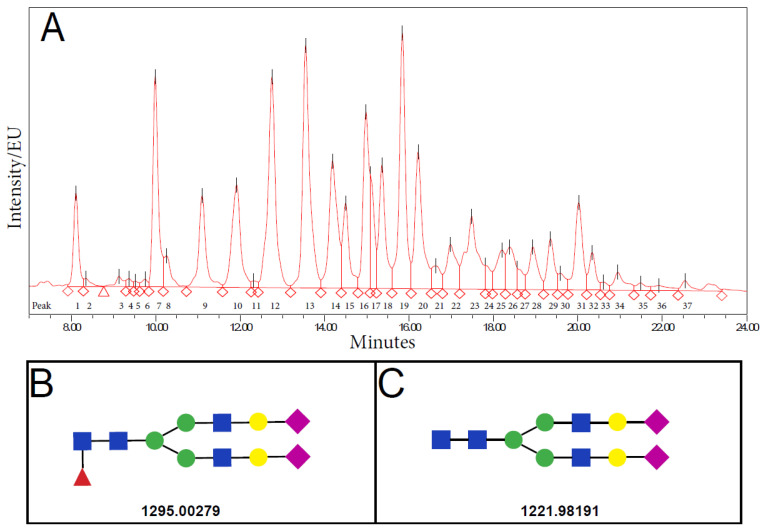
(**A**) Representative chromatogram of N-glycans isolated from seminal plasma total proteins determined by ultra-high-performance liquid chromatography based on hydrophilic interactions (HILIC-UHPLC). EU = emission units. Structures of glycans identified in the glycan peak SPGP14 are biantennary digalactosylated disialylated glycan with (**B**) and (**C**) without core fucose.

Table 3 and Table 4 show the relative abundance of 37 individual seminal plasma N-glycan peaks (SPGP) for all studied groups, and Figure 3 shows a representative chromatogram. Results show that the N-glycan relative area of SPGP14 and SPGP27 significantly differed between the normozoospermic and pathological groups, with the SPGP14 relative area being significantly reduced while SPGP27 increased in the group with pathological semen parameters. However, following a Bonferroni correction, only the change in SPGP14 remained statistically significant (Table 5).

Following adjustment of the log-normal regression model for confounders, including age, DFI, and sperm chromatin maturity, the statistical model that compares the N-glycans peaks between the normozoospermic and pathological groups confirms the results but also delivers novelties as shown in Table 5. The most striking observation was again obtained for the SPGP14 relative area, which shows a 16% (*p* = 0.001) reduction in the pathological group. Even in the multivariate setting, SPGP14 was the only glycan peak with an adjusted *p*-value less than 0.05 (Table 5). 

In Table 5, the A, O, and OA sub-groups significantly differed, in at least one group compared to normal group for SPGP2, SPGP4, SPGP6, SPGP14, SPGP18, SPGP26, and SPGP35 relative area, however without a clear trend between sub-groups. Again, SPGP14 was the only N-glycan that remained significantly changed between normozoospermic and all three pathological sub-groups after the Bonferroni correction. The OA group had the greatest reduction in SPGP14 area values (3.13 ± 0.86), followed by O (3.39 ± 1.50) and A (3.89 ± 1.33) groups. Concerning the significance of the reducing trend, the reduction in A (−5%, *p* = 0.409) was not significant compared to normozoospermic men; however, significant reduction was detected in O (−19%, *p* = 0.004) and OA (−26%, *p* < 0.001) group (Table 5). Furthermore, we performed a pairwise comparison between all groups to evaluate the differences in the area of specific N-glycans reported in Table 5 for all groups. After applying the Bonferroni correction for multiple comparisons, we found that SPGP14 area values were significantly higher in the A group than in the OA group. The glycans SPGP2 and SPGP4 were present in the O group at significantly greater quantities than in the OA group, while SPGP18 was significantly higher in the OA group with respect to the A group.

### 2.5. Structural Characterization of SPGP14

Structural characterization of glycans eluting in SPGP14 was performed by liquid chromatography-mass spectrometry (LC-MS). The identified glycans were with the composition H5N4F1S2 (*m/z* 1295.00279) and H5N4S2 (*m/z* 1221.98191) as the most abundant in the peak. These compositions would correspond to complex-type glycans biantennary digalactosylated disialylated glycan with (FA2G2S2) and without core fucose (A2G2S2), respectively (Figure 3B,C).

### 2.6. Association of N-Glycans with Semen Parameters

Considering other potentially relevant N-glycans, the results are reported for all N-glycan peaks that differed significantly in at least one pathological sub-group from the normozoospermic group. The relative peak area of SPGP6 showed a clear downward trend, a reduction by 18% (*p* = 0.015) in the pathological groups, while SPGP26 showed a 21% (*p* = 0.026) increase in the pathological group. The positive trend for SPGP26 was exclusively observed in the A (+33%, *p* = 0.009) and O (+28%, *p* = 0.043) groups (Table 5), although it did not remain significant after Bonferroni’s correction. There was no clear trend for the other N-glycan peaks and pathological semen parameters. Correlation of seminal plasma N-glycan peaks and individual semen parameters was not observed.

### 2.7. Association between N-Glycans and Sperm Chromatin Maturity 

The multivariate analysis showed a significant correlation between the percentage of sperm with mature chromatin and N-glycan relative area for SPGP17 and SPGP25. When the percentage of mature sperm increased by 10%, a reduction in SPGP17 relative area by 14% (*p* = 0.037) was found, while SPGP25 increased by 25% (*p* = 0.034). 

The diagnostic tests applied to verify the efficacy of the statistical model did not reveal any violations of the log-normal regression model’s assumptions.

## 3. Discussion

Spermatogenesis is a complex, tightly spatiotemporally regulated process of male gamete maturation from self-renewing undifferentiated spermatogonia through haploid spermatids to spermatozoa and finally mature sperm. Disturbances in any of the developmental stages of sperm due to various reasons may cause irreversible damage resulting in male reproductive disorders and infertility [33,34]. Recently, a systematic analysis conducted by Sun et al., 2019, emphasized that male infertility prevalence rises annually across the globe [35]. Therefore, early detection and prediction of male reproductive dysfunction beyond standard semen analysis could improve the current management of male infertility [36]. 

In the present study, our results first show that SDF was significantly increased in the pathological groups and was inversely correlated with sperm motility and viability. Second, SPGP14, which corresponds to complex-type glycans, biantennary digalactosylated disialylated glycan with (FA2G2S2) and without core fucose (A2G2S2), significantly differs between men with normal and pathological semen parameters. Finally, sperm chromatin maturity is shown to be significantly associated with SPGP17 and SPGP25. Moreover, an important finding is that seminal plasma glycan patterns significantly differ between A, O, and OA subgroups, suggesting that glycans might represent a promising future biomarker candidate in male infertility diagnostics. 

Genome damage has been previously negatively linked with fertilization, embryo quality, and implantation rates [37]. Even though unequivocal cut-off values for DFI are still not standardized, a recent meta-analysis on 2883 infertile (mean age 35.22 ± 4.31 years) and 1294 fertile men (mean age 34.24 ± 3.03 years) assessed an SDF threshold value of 20% with 79% sensitivity and 86% specificity [38]. Our results fit perfectly within the proposed threshold since in our study normozoospermic men had an average DFI below 20%, while all pathological groups had an average DFI above the mentioned value (Table 1 and Table 2).

Moreover, we observed that SDF significantly increases with the disruption of sperm parameters, showing the highest prevalence in men with reduced sperm count and motility (OA), but implying a stronger negative correlation of sperm genome damage with disruption in motility rather than concentration. These results are in concordance with the majority of the previously published studies on the clinical utility of DNA fragmentation [39,40,41,42], with a potential explanation suggesting that motility and SDF both originate during spermiogenesis and are susceptible to oxidative stress [43,44]. However, some studies do not show preferential correlation with either sperm count, motility, or morphology [45,46], or associated sperm concentration to SDF [47]. These discrepancies could arise from the different or non-standardized methodology for assessing sperm genome damage, as considerable inter-laboratory variability exists, but also from unclear threshold setups for various types of DNA fragmentation assays [48]. Moreover, our results showed that SDF is also inversely correlated with sperm viability; hence, men with viability values above the WHO threshold value (≥58%) probably do not benefit from sperm DNA fragmentation testing [49]. 

N-glycosylation, one of the most prevalent post-translational protein modifications, plays a significant role in interactions between cells and extracellular matrix as well as in protein folding. The glycosylation pattern was shown to be altered in various diseases and conditions, such as male infertility [13], where the seminal plasma has been extensively explored, as it offers a rich source of N-glyco-biomarkers [50]. Although seminal plasma surrounds sperm during a short period, protein glycosylation is stable under physiological conditions and can have a significant impact on protein function, as many adhesive properties result from the protein–carbohydrate interactions. These properties are especially important for proper sperm activation and successful fertilization [13] since membrane glycoproteins of sperm glycocalyx directly interact with seminal plasma glycoproteins, which affects sperm behavior regarding premature capacitation and immune suppression in the female reproductive tract [51,52,53]. Recently, a glycoprotein clusterin (CLU), one of the crucial seminal plasma glycoproteins, has been proposed as a male infertility marker, due to its significant role in sperm capacitation and immune tolerance in the female reproductive tract. However, despite many new scientific reports concerning seminal plasma glycoproteins and its relation to sperm parameters, a new biomarker indicating male fertility disorders is still warranted [54].

In the present study, we propose a relative area of seminal plasma glycans peak SPGP14 as a candidate biomarker for the separation of men with normal and pathological semen parameters. Composition analysis of SPGP14 identified biantennary digalactosylated disialylated glycan with (FA2G2S2) and without core fucose (A2G2S2) as the most abundant in the peak. The N-glycans found in our study were for the first time associated with male infertility [15,17,55]. Previous studies that identified N-glycans from seminal plasma of fertile donors found highly fucosylated complex type N-glycans with varying levels of sialylation and core fucosylation, antennary fucosylation, or both [15,17]. Fucosylation of seminal plasma glycans seems to be better studied or interpreted at an antennary location, where it is a part of Lewis epitopes involved in the process of fertilization, than in the core region of seminal plasma glycans [56]. Moreover, antennary fucosylation may be a common and important trait of seminal plasma glycoproteins, and hyperfucosylation may influence their interactions. The proteins that carry these critical glycan modifications could serve as helpful glycomarkers in the examination of semen status [56]. Differences in highly branched N-glycans were also found between fertile and infertile men [21]. Glycosylation was also investigated in specific seminal plasma proteins. As prostate specific antigen (PSA) is one of the most abundant glycoproteins of seminal plasma, Wang et al., recently found that the most abundant glycoform on isolated PSA from seminal plasma is H5N4F1S2, and α2,6-sialylation the most abundant linkage variant [57]. Surprisingly, the same glycans were found in our glycan peak SPGP14, which could indicate that glycosylation differences could be at least partially due to PSA, although the authors did not find differences in this particular glycan between fertile and infertile men [57]. Another study investigated the concentration and glycosylation pattern of glycoprotein CLU in serum and seminal plasma, suggesting that serum CLU concentration and core fucosylation distinguished between normozoospermic group and group with abnormal sperm count, motility and morphology. However, the seminal plasma CLU did not show the same pattern or the correlations with semen parameters [58]. The differences between specific glycan modifications were observed between fertile and infertile men, including diminished sialylation, with the most prominent in asthenozoospermic men [22]. Furthermore, the α2,3-linked sialic acid was previously found to be significantly different in asthenozoospermic men compared to all other groups [59]. 

Another significant result in our study is that astheno-, oligo-, and oligoasthenozoospermic pathological sub-groups did not share the same glycosylation profile deviations. Different N-glycan patterns of several SPGPs, namely SPGP2, SPGP4, SPGP6, SPGP14, SPGP18, SPGP26, and SPGP35, were observed between normozoospermic and pathological groups. These differences in the N-glycan patterns may potentially contribute to hemostasis of sperm and seminal plasma, but also indicate changes or impairment in the glycosylation process that occurs in seminal vesicles or other secretory glands [55]. Further elucidation of seminal plasma N-glycans could potentially highlight some of them as markers of specific sperm pathology.

Histone retention measured by AB was shown to be a suitable predictor of ART outcomes, including embryo quality and clinical pregnancy [60]. However, the reliability of AB in remaining histone level assessments as the measurement of sperm chromatin maturity is still a contradictory and better interpretation of results taking into account that other measured parameters is warranted [31,61,62]. The correlation between the percentage of AB-stained spermatozoa and sperm parameters remains controversial. Mature sperm chromatin may or may not correlate with asthenozoospermic samples and abnormal morphology patterns [31,61,63,64]. Hammadeh et al., 2001 reported a significant difference between patients and healthy donors, but no correlation was found between sperm chromatin maturity and morphology, count or motility [62]. These inconsistent results potentially indicate an independence of sperm chromatin maturity as a parameter in male infertility evaluation. 

Nonetheless, we showed a significant association between sperm chromatin maturity and glycan peaks SPGP17 and SPGP25 for the first time, suggesting a possible new mechanism involved in the etiology of infertility. Here, we imply that the introduction of SPGPs in the interpretation of the AB results could potentially upraise the predictivity of successful fertilization. At the same time, glycan peaks may enhance interpretation of the AB results alone, since our study demonstrated the lack of significant association between histone retention, determined by AB staining, with sperm parameters and DNA fragmentation. This lack or weak correlation between sperm DNA fragmentation and different assays assessing sperm chromatin maturity was previously observed [65]. Other factors than defective sperm chromatin remodeling, such as oxidative stress or meiotic breaks during spermatogenesis, also contribute to DNA fragmentation; hence, it is not unexpected that sperm chromatin maturity is necessarily correlated with SDF [66]. Moreover, the proper condensation of sperm chromatin heavily depends on protamine content, quality of disulfide bonds between the protamine rings, and the zink bioaviability [67,68,69]. On the other hand, the exact molecular explanation of association of N-glycans from seminal plasma with an expression of histones or protamines in spermatozoa after spermiogenesis remains unclear. A study by Girouard et al. showed that parts of epididymal bovine spermatozoa plasma membrane could be reorganized by specific seminal plasma proteins [70]. Potentially, signaling molecules of sperm plasma membrane, which could depend on the chromatin status of mature sperm, interact differently with the glycoproteins from seminal plasma. Thus, SPGP17 and SPGP25 may be suggested as predictors of sperm chromatin status, and further studies are necessary to elucidate the function of these N-glycans.

Sperm may be defined as a life capsule with self-sustainable biological support. Consequently, crossroads of pathways that take part in its architecture and functionality are crucial in diagnostics and investigation. The main measured sperm characteristics that categorize men as infertile remain count, motility, morphology, sperm DNA integrity, and chromatin maturity. Still, the complexity of male infertility demands a simultaneous analysis of multiple parameters that can recognize major pathways crucial in the etiology of infertility but also in the personalized approach to sperm contribution in ART. In the sense of that glycan peaks observed here could serve as one of the promising candidates in infertility biomarkers research that should be further explored. 

## 4. Materials and Methods

### 4.1. Study Sample Collection

All procedures were carried out according to the regulations of the Declaration of Helsinki. The Ethics Committee and University Hospital Zagreb, Croatia, approved this study with the reference number 021-1/41-18 and approval date 15 March 2018. Study participants were recruited at the Clinical Department for Laboratory Diagnostics, University Hospital Zagreb, Croatia, after signing voluntarily informed consent. The study group was between 20–55 years old and referred to semen analysis after examination by an andrologist. Exclusion criteria were: (1) absence of sperm in the neat ejaculate and (2) previous chemo- or radiation therapy. Samples were collected into sterile containers by masturbation after 2–7 days of abstinence and processed after 30–60 min from liquefaction. Analysis of semen samples was conducted according to World Health Organization (2010) criteria for semen parameter evaluation. The samples were classified as normozoospermic when semen volume was above 1.5 mL, sperm total motility was 40% or above, progressive motility was ≥32%, sperm concentration was ≥15 × 10^6^ spermatozoa per mL or ≥39 × 10^6^ spermatozoa per ejaculate, and morphology was ≥4% [6,32]. If any of the semen parameters were below the reference limits, the samples were categorized as pathological. Finally, semen samples were obtained from a total of 166 participants with 82 normozoospermic and 84 pathological diagnoses. Pathological samples were further subcategorized according to concentration and motility parameters into asthenozoospermic (*n* = 29), oligozoospermic (*n* = 21), and oligoasthenozoospermic (*n* = 34).

### 4.2. Semen Parameter Analysis by CASA (Computer-Assisted Semen Analysis)

After liquefaction at room temperature (RT), ejaculates were well mixed, seminal volume and pH were measured, and 10 µL of semen samples was transferred to a Makler counting chamber (FujiFilm Irvine Scientific, Santa Ana, CA, USA). Sperm concentration and motility were analyzed using the automated SCA^®^ system (Sperm Class Analyser, CASA System, Microptic SL, Barcelona, Spain), and output data were obtained in the Motility module of SCA evolution software (Microptic SL, Barcelona, Spain). Sperm concentration was reported as count per ml and a total number of spermatozoa in the volume of ejaculate, while motility was determined as a percentage of rapid, slow progressive and non-progressive spermatozoa. Subsequently, spermatozoa smears were fixed in methanol for 5 min at RT, incubated for 20 min with Giemsa dye, and mounted. Sperm morphology data was obtained with the Morphology module of SCA evolution software.

### 4.3. DNA Fragmentation Assay

Sperm DNA fragmentation was determined using a sperm chromatin dispersion (SCD) test (GoldCyto Sperm ^®^Kit, Goldcyto Biotech Corp., Guangzhou, China). Samples were diluted at concentration 5 million per ml with PBS, and 30 µL of each sample was resuspended in dissolved agarose. On the pretreated slide, 20 µL of the sample was added and covered with a coverslip. Slides were then incubated at +4 °C for 10 min and kept at a horizontal position throughout the entire process. Slides were incubated with acidic denaturation solution for 7 min and with lysis solution for 25 min at RT. Next, slides were rinsed in distilled water for 5 min and washed in 70%, 90%, and 100% ethanol for 2 min at RT. After drying, slides were stained with 500 µL of TA solution for 1 min. Then, 1500 µL of TB solution was added directly on a slide with TA solution, gently mixed and incubated for another 10 min. Slides were then rinsed with abundant distilled water and dried. Thirty frames per sample were evaluated on CASA with DNA fragmentation module on SCA evolution software (Microptic SL, Barcelona, Spain). Spermatozoa showing big and medium-size halos were considered as without DNA fragmentation, while spermatozoa with small and no halos were considered as fragmented.

### 4.4. Aniline Blue Assay

Sperm chromatin maturity was evaluated with acidic aniline blue (AB) staining, which binds to remaining histones in the sperm nucleus. Semen samples were diluted if necessary to 10 million sperm per ml, the smear was made with 20 µL of sample and allowed to air dry. Next, samples were fixed for 30 min with 3% glutaraldehyde in phosphate-buffered saline (PBS, pH = 7.4) at RT. Samples were then washed with PBS and dH_2_O, 10 min each at RT. Samples were air-dried and stained with 5% aqueous aniline blue solution mixed with 4% acetic acid (pH = 3.5) for 5 min at RT. Slides were mounted in anisole and analyzed with a light microscope under magnification 100×. For each smear, 100 spermatozoa were evaluated as stained or unstained. Stained spermatozoa were considered immature and abnormal, and spermatozoa with light to no staining were considered normal.

### 4.5. N-Glycan Analysis from Total Seminal Plasma Proteins

Seminal plasma was separated from cellular components by centrifugation. Briefly, samples were resuspended and aliquoted in 1.5 mL tubes and centrifuged for 10 min at 650 rpm at RT. Seminal plasma was further centrifuged at 3550 rpm and stored at −80 °C until further use. In-house standard of seminal plasma was prepared by pooling 5 μL of each seminal plasma sample and used as a control throughout the sample preparation procedure. N-glycans from total seminal plasma proteins were isolated using a 96-well plate, starting with 5 µL of the sample. Then, 200 μL of 50 mg/mL Chromabond C-18 beads (Marcherey-Nagel, Düren, Germany) suspension in acetonitrile (ACN, Carlo Erba, Cornaredo, Italy)/0.1% trifluoroacetic acid (TFA, Sigma-Aldrich, St. Louis, MI, USA) (80:20) was pipetted to each well of an Orochem plate (Orochem, Naperville, IL, USA). The beads were then washed using a vacuum manifold (Pall Corporation, New York, NY, USA) three times with 200 µL ACN/0.1% TFA (80:20), and three times with 200 µL ACN/0.1% TFA (5:95). Samples were diluted with 45 µL of ACN/0.1% TFA (5:95), transferred to the plate, and 150 µL of ACN/0.1% TFA (5:95) was further added to each sample on the plate. After two-minute incubation free glycans were removed by plate centrifugation for 5 min with increasing speed from 300 to 800 rpm (centrifuge 5804 with a rotor A-2-DWP, Eppendorf, Germany) Glycoproteins were eluted from the C-18 beads by adding 200 μL of 80% ACN to each well of the Orochem plate, incubation for two minutes, and repeating the centrifugation step. Glycoprotein eluate was collected in a PCR plate (Thermo Fisher Scientific, Waltham, MA, USA) and dried overnight at 37 °C. The next day, proteins were denatured with 30 µL of 1.33% SDS (Carl Roth, Karlsruhe, Germany) and incubation for 10 min at 65 °C. Samples were then treated with 4% Igepal CA-630 (Sigma-Aldrich), 10 µL for 30 min. Then, 1.25 mU PNGase F (Promega, Madison, WI, USA) in 10 µL of 5 × PBS was added to each sample and incubated at 37 °C for 18 h. 

Released N-glycans in samples were labeled with 25 µL of procainamide (Sigma-Aldrich) mixture containing 4.32 mg of procainamide in glacial acetic acid (Honeywell, Charlotte, NC, USA)/dimethylsulfoxide (Sigma-Aldrich) (30:70) per sample. The plate containing samples was sealed with an adhesive seal and incubated at 65 °C for 1 h. Reducing agent solution, made by dissolving 4.48 mg 2-picoline borane (J&K Scientific, Beijing, China) in 25 µL of glacial acetic acid/dimethylsulfoxide (30:70) per sample, was further added to the samples and incubated for an additional 1.5 h at 65 °C. Samples were then cooled for 30 min at RT. Labeled N-glycans were cleaned up as previously described [71], with a modification of using 0.2 μm Supor AcroPrep filter plate (Pall Corporation) as a stationary phase.

### 4.6. Detection and Measurement of N-Glycans 

Fluorescently labeled N-glycans were separated by hydrophilic interaction liquid chromatography (HILIC) on a Waters Acquity ultra-high-performance liquid chromatography (UHPLC) H-class UPLC instrument (Milford, MA, USA) consisting of a quaternary solvent manager, sample manager, and a fluorescence detector with excitation and emission wavelengths of 310 and 370 nm, respectively. The separation of N-glycans was conducted at 25 °C using a 2.1 × 150 mm ACQUITY UPLC Glycan BEH Amide column (Waters, 1.7 µm particle size, 130 Å pore size) at a flow rate of 0.561 mL/min. The sample temperature was 10 °C. Mobile phase A was 100 mM ammonium formate in water (pH 4.4), while mobile phase B was 100% ACN. The samples were eluted with a linear gradient from 30% to 47% solvent A over 23.34 min after the initial isocratic step of 30% solvent A for 1.47 min. The instrument was controlled by Empower 3 software, build 3471 (Waters). Data were processed using an automatic processing method with a traditional integration algorithm, and each chromatogram was manually corrected to maintain the same intervals of integration for all samples. All chromatograms were separated into 37 individual seminal plasma N-glycan peaks (SPGP1-SPGP37), and N-glycan values were expressed as a percentage of the total integrated area (% area values).

### 4.7. Structural Characterization of SPGP14

For structural characterization of glycans eluted in SPGP14, the released and labeled N-glycans of prepared in-house seminal plasma standard were analyzed by liquid chromatography-mass spectrometry (LC-MS) on a BioAccord LC-MS System (Waters). Chromatographic conditions were the same as described above. The RDa mass detector was used in-line via electrospray ionization in positive mode. The settings were as follows: scan range, 50–2000 *m*/*z*; capillary voltage, 1.5 kV; cone voltage, 45 V; desolvation temperature, 300 °C; and sampling rate, 2 Hz. Acquired data were automatically processed using the UNIFI 1.9.9.3 Scientific Information System. For the glycan identification, MS^1^ sum spectra were generated around the retention times (RT) of the chromatographic peak reported as SPGP14 (RT 12.99–13.33 min). Annotation of MS^1^ sum spectra was inferred from the *m/z* values and deduced using GlycoMod software (https://web.expasy.org/glycomod/, accessed on 7 July 2021). MS interpretation was based on previously reported structures [15] and biosynthetic pathways.

### 4.8. Statistical Analysis

As for the descriptive analysis, the non-parametric Mann–Whitney test was used to compare the normozoospermic men versus the men with pathological diagnoses. When the four groups chosen based on sperm count and motility were analyzed—normozoospermic, asthenozoospermic, oligozoospermic and oligoasthenozoospermic—the Kruskal–Wallis test was performed. The log-normal regression model was applied to investigate the relationship between N-glycans and infertility adjusted by age, DNA fragmentation, and staining. This statistical model allows estimation of change in the mean frequency of the N-glycans in each group, while compared to the normozoospermic group. To verify if the assumptions of the log-normal model were violated, the residual-versus-fitted plot and the test for heteroskedasticity were performed. Since 37 N-glycan peaks were tested simultaneously, Bonferroni’s correction for multiple comparisons was applied. STATA software (StataCorp. 2015. Stata: Release 14.2. Statistical Software, College Station, TX, USA: StataCorp LP) was used for all analyses.

## 5. Conclusions

In conclusion, we confirmed previous studies that indicate the importance of sperm DNA fragmentation testing and the strong inverse correlation of DFI and sperm motility and viability. Additionally, we detected the novel total protein seminal plasma N-glycan peak SPGP14, whose reduction showed a significant tendency towards pathological semen parameters. Furthermore, we demonstrated the association between sperm chromatin maturity and glycan composition, including SPGP17 reduction and SPGP25 increase with a higher percentage of sperm with mature chromatin. In this work, we pointed out the detection of independent glycan peak SPGP14 that can further assist male infertility diagnostics, but we also introduced the combined performance of classical AB staining with seminal plasma glycan profiling as a potential contributor to improved understanding and management of male infertility diagnostics. In addition, the presence of seminal plasma glycans specific for A, O, and OA sub-diagnosis as well as a more in-depth understanding of interaction between sperm quality and seminal plasma require further investigation regarding their clinical potential as biomarkers of certain male infertility disorders.

## Figures and Tables

**Table 1:** Descriptive analysis of study participants diagnosed with normozoospermic (N) and pathological (P) semen values. *p*-value was determined by using the Mann–Whitney test.

	N (*N* = 82)		P (*N* = 84)		*p*-Value
	Mean	SD	Mean	SD	
Age	34.5	6.4	36.0	6.8	0.178
Sperm concentration (10^6^/mL)	59.1	29.0	33.4	92.9	<0.001
Total sperm count (10^6^/ejaculate)	188.3	116.1	72.4	115.4	<0.001
Volume (mL)	3.3	1.3	3.2	1.8	0.269
DFI (%)	17.8	9.2	30.7	17.5	<0.001
Big halo sperm (%)	58.1	18.7	42.1	20.3	<0.001
Medium halo sperm (%)	24.1	15.4	27.4	12.3	0.010
Small halo sperm (%)	3.6	2.1	5.4	5.3	0.019
Sperm without halo (%)	13.6	8.4	24.7	14.2	<0.001
Degraded sperm (%)	0.6	0.8	0.6	0.8	0.421
Sperm chromatin maturity (%)	94.5	4.8	95.4	4.1	0.197

DFI–DNA fragmentation index.

**Table 2:** Descriptive analysis to compare men diagnosed with normozoospermic (N) with respect to pathological semen parameters separated into astheno-(A), oligo-(O) and oligoasthenozoospermia (OA). *p*-value determined by using the Kruskal–Wallis test. A nominal *p*-value less than 0.05 means that at least one group significantly differs from the others.

	N (*N* = 82)		A (*N* = 29)		O (*N* = 21)		OA (*N* = 34)		*p*-Value
	Mean	SD	Mean	SD	Mean	SD	Mean	SD	
Age	34.5	6.4	36.0	7.7	37.0	6.7	35.4	6.0	0.516
Sperm concentration (10^6^/mL)	59.1	29.0	52.7	29.8	12.1	10.6	30.2	142.3	<0.001
Total sperm count (10^6^/ejaculate)	188.3	116.1	165.1	158.4	26.3	19.2	21.8	21.2	<0.001
Volume (mL)	3.3	1.3	3.0	1.3	2.7	1.4	3.7	2.2	0.229
DFI (%)	17.8	9.2	27.6	15.3	21.6	7.2	38.9	20.3	<0.001
Big halo sperm (%)	58.1	18.7	49.3	20.4	49.6	15.8	31.3	18.3	<0.001
Medium halo sperm (%)	24.1	15.4	23.5	10.3	28.8	12.5	29.8	13.3	0.019
Small halo sperm (%)	3.6	2.1	4.9	4.6	3.6	2.0	7.1	6.6	0.005
Sperm without halo (%)	13.6	8.4	22.1	12.2	17.7	6.3	31.1	16.7	<0.001
Degraded sperm (%)	0.6	0.8	0.7	0.8	0.3	0.4	0.7	1.0	0.164
Sperm chromatin maturity (%)	94.5	4.8	94.7	4.0	96.2	4.6	95.5	3.8	0.216

DFI–DNA fragmentation index.

**Table 3:** Descriptive analysis of mean N-glycan relative area in men diagnosed with normozoospermic and pathological semen parameters. *p*-value determined by using the Mann–Whitney test.

Seminal Plasma Glycan Peak (SPGP)	N (*N* = 82)		P (*N* = 84)		Nominal *p*-Value
	Mean	SD	Mean	SD	
SPGP1	2.27	0.40	2.35	0.65	0.989
SPGP2	0.23	0.09	0.23	0.10	0.406
SPGP3	0.32	0.12	0.34	0.11	0.307
SPGP4	0.21	0.08	0.22	0.10	0.887
SPGP5	0.11	0.07	0.12	0.06	0.336
SPGP6	0.16	0.08	0.14	0.07	0.085
SPGP7	4.94	1.95	4.98	2.24	0.957
SPGP8	0.85	1.03	0.71	0.27	0.384
SPGP9	2.87	0.85	2.90	1.10	0.992
SPGP10	3.03	0.66	3.07	0.93	0.744
SPGP11	0.19	0.43	0.16	0.18	0.961
SPGP12	7.18	1.70	7.26	2.16	0.906
SPGP13	6.93	1.21	6.80	1.26	0.579
SPGP14 *	**3.85**	**0.89**	**3.46**	**1.24**	**<0.001**
SPGP15	11.36	7.46	12.68	7.89	0.150
SPGP16	3.81	1.12	3.65	1.33	0.167
SPGP17	2.78	1.47	2.79	1.23	0.791
SPGP18	9.73	3.56	9.60	4.05	0.855
SPGP19	5.40	2.41	5.38	2.10	0.899
SPGP20	7.46	4.39	7.04	3.85	0.492
SPGP21	1.16	0.59	1.13	0.53	0.952
SPGP22	2.15	1.21	2.17	1.35	0.960
SPGP23	3.65	1.91	3.46	2.00	0.217
SPGP24	2.11	1.86	2.18	1.79	0.497
SPGP25	1.43	0.96	1.31	0.82	0.349
SPGP26	1.19	0.77	1.39	1.02	0.134
**SPGP27**	**1.38**	**0.61**	**1.59**	**0.64**	**0.018**
SPGP28	1.74	0.71	1.79	0.85	0.997
SPGP29	2.40	1.05	2.48	1.14	0.896
SPGP30	1.12	0.83	1.00	0.55	0.548
SPGP31	2.89	1.74	2.72	1.80	0.436
SPGP32	1.23	0.52	1.19	0.66	0.063
SPGP33	0.74	0.49	0.71	0.47	0.906
SPGP34	0.77	0.37	0.85	0.52	0.426
SPGP35	0.49	0.30	0.44	0.25	0.423
SPGP36	0.85	0.72	0.81	0.51	0.745
SPGP37	1.00	0.53	0.90	0.43	0.294

*: *p* < 0.05 after Bonferroni correction.

**Table 4:** Descriptive analysis of mean relative to compare N-glycan area in men diagnosed with normozoospermic (N) with respect to pathological semen values separated into astheno (A)-, oligo (O)-, and oligoasthenozoospermia (OA). *p*-value determined by using the Kruskal–Wallis test. A nominal *p*-value less than 0.05 means that at least one group significantly differs from the others for that glycan.

Seminal Plasma Glycan Peak (SPGP)	N (*N* = 82)		A (*N* = 29)		O (*N* = 21)		OA (*N* = 34)		Nominal *p*-Value
	Mean	SD	Mean	SD	Mean	SD	Mean	SD	
SPGP1	2.27	0.40	2.30	0.52	2.35	0.89	2.40	0.60	0.676
SPGP2	0.23	0.09	0.22	0.10	0.25	0.09	0.22	0.10	0.183
SPGP3	0.32	0.12	0.35	0.14	0.36	0.09	0.31	0.09	0.112
SPGP4	0.21	0.08	0.23	0.13	0.25	0.10	0.19	0.07	0.117
**SPGP5**	**0.11**	**0.07**	**0.10**	**0.05**	**0.14**	**0.04**	**0.11**	**0.08**	**0.042**
SPGP6	0.16	0.08	0.16	0.09	0.14	0.05	0.13	0.06	0.168
SPGP7	4.94	1.95	4.31	1.95	5.34	2.53	5.33	2.23	0.135
SPGP8	0.85	1.03	0.64	0.21	0.72	0.28	0.75	0.30	0.314
SPGP9	2.87	0.85	3.00	1.22	3.04	1.43	2.73	0.72	0.851
SPGP10	3.03	0.66	2.95	0.64	3.37	1.52	2.99	0.61	0.386
SPGP11	0.19	0.43	0.13	0.08	0.22	0.33	0.16	0.07	0.244
SPGP12	7.18	1.70	7.68	2.36	7.31	2.62	6.87	1.60	0.670
SPGP13	6.93	1.21	6.70	1.37	6.82	1.14	6.87	1.26	0.655
SPGP14 *	**3.85**	**0.89**	**3.89**	**1.33**	**3.39**	**1.50**	**3.13**	**0.86**	**<0.001**
SPGP15	11.36	7.46	13.84	9.19	11.99	7.79	12.11	6.81	0.499
SPGP16	3.81	1.12	3.54	1.17	3.67	1.44	3.73	1.41	0.553
**SPGP17**	**2.78**	**1.47**	**3.12**	**1.30**	**3.19**	**1.47**	**2.27**	**0.78**	**0.032**
SPGP18	9.73	3.56	8.58	3.72	9.46	3.37	10.55	4.55	0.348
SPGP19	5.40	2.41	5.04	2.38	5.09	1.98	5.84	1.88	0.387
SPGP20	7.46	4.39	6.21	3.76	6.62	3.53	8.01	4.00	0.442
SPGP21	1.16	0.59	1.16	0.45	1.19	0.73	1.07	0.44	0.801
SPGP22	2.15	1.21	2.25	1.60	2.25	1.57	2.06	0.94	0.950
SPGP23	3.65	1.91	4.13	2.22	3.01	1.59	3.17	1.93	0.071
SPGP24	2.11	1.86	2.21	1.74	2.20	1.52	2.14	2.01	0.770
SPGP25	1.43	0.96	1.39	0.84	1.31	0.91	1.23	0.75	0.632
**SPGP26**	**1.19**	**0.77**	**1.51**	**0.76**	**1.70**	**1.66**	**1.09**	**0.54**	**0.012**
SPGP27	1.38	0.61	1.55	0.70	1.55	0.64	1.65	0.60	0.086
SPGP28	1.74	0.71	1.58	0.71	1.98	0.93	1.86	0.90	0.478
SPGP29	2.40	1.05	2.66	1.50	2.36	1.05	2.40	0.79	0.974
SPGP30	1.12	0.83	1.01	0.49	0.97	0.51	1.00	0.63	0.609
SPGP31	2.89	1.74	2.39	1.37	2.74	1.21	3.00	2.34	0.436
**SPGP32**	**1.23**	**0.52**	**1.38**	**0.69**	**1.07**	**0.46**	**1.09**	**0.72**	**0.035**
SPGP33	0.74	0.49	0.77	0.47	0.67	0.41	0.70	0.50	0.887
SPGP34	0.77	0.37	0.82	0.41	1.02	0.64	0.77	0.51	0.420
SPGP35	0.49	0.30	0.42	0.26	0.49	0.18	0.43	0.28	0.314
SPGP36	0.85	0.72	0.84	0.45	0.88	0.70	0.74	0.44	0.748
SPGP37	1.00	0.53	0.94	0.46	0.88	0.44	0.89	0.41	0.712

*: *p* < 0.05 after Bonferroni correction.

**Table 5:** Log-normal regression models to compare N-glycan area in men diagnosed with normozoospermic (N) versus pathological semen values separated into astheno (A)-, oligo (O)-, and oligoasthenozoospermia (OA). Percentage changes were adjusted by age, DNA fragmentation, and sperm chromatin maturity.

Seminal Plasma Glycan Peak (SPGP)	Change (%) in the Mean Relative Area of N-Glycans		
	A	O	OA
SPGP2	−14% (0.079)	+8% (0.399)	−**22% (0.008)**
SPGP4	−2% (0.818)	+11% (0.258)	−**22% (0.003)**
SPGP6	−8% (0.406)	−18% (0.105)	−**30% (0.002)**
SPGP14 *	−5% (0.409)	−**19% (0.004)**	−**26% (<0.001)**
SPGP18	−7% (0.433)	+3% (0.769)	**+29% (0.012)**
SPGP26	**+33% (0.009)**	**+28% (0.043)**	0% (0.978)
SPGP35	−**25% (0.035)**	+10% (0.524)	−**27% (0.033)**

*: *p* < 0.05 after Bonferroni correction. A—asthenozoospermia, O—oligozoospermia, OA—oligoasthenozoospermia.

## Data Availability

Not applicable.
